# *Ceratopteris richardii* (C-fern): a model for investigating adaptive modification of vascular plant cell walls

**DOI:** 10.3389/fpls.2013.00367

**Published:** 2013-09-23

**Authors:** Olivier Leroux, Sharon Eeckhout, Ronald L. L. Viane, Zoë A. Popper

**Affiliations:** ^1^Botany and Plant Science and The Ryan Institute for Environmental, Marine and Energy Research, School of Natural Sciences, National University of IrelandGalway, Ireland; ^2^Department of Biology, Research Group Pteridology, Ghent UniversityGhent, Belgium

**Keywords:** plant cell wall, ferns, vascular plants, monoclonal antibodies, development, tissue-specificity, mannans

## Abstract

Plant cell walls are essential for most aspects of plant growth, development, and survival, including cell division, expansive cell growth, cell-cell communication, biomechanical properties, and stress responses. Therefore, characterizing cell wall diversity contributes to our overall understanding of plant evolution and development. Recent biochemical analyses, concomitantly with whole genome sequencing of plants located at pivotal points in plant phylogeny, have helped distinguish between homologous characters and those which might be more derived. Most plant lineages now have at least one fully sequenced representative and although genome sequences for fern species are in progress they are not yet available for this group. Ferns offer key advantages for the study of developmental processes leading to vascularisation and complex organs as well as the specific differences between diploid sporophyte tissues and haploid gametophyte tissues and the interplay between them. *Ceratopteris richardii* has been well investigated building a body of knowledge which combined with the genomic and biochemical information available for other plants will progress our understanding of wall diversity and its impact on evolution and development.

## INTRODUCTION

Driven by an increased awareness of the impact of plant cell wall composition on environmental responses, and their commercial exploitation, as well as by curiosity, and facilitated by technological developments, cell wall diversity and evolution has increasingly become a major research focus in the last 5 years (Popper, 2008; Sarkar et al., 2009; Yin et al., 2009; Popper and Tuohy, 2010; Sørensen et al., 2010; Popper et al., 2011; Fangel et al., 2012). Cell walls are involved at every level of plant morphology, growth and development, and have changed during evolution (Popper and Fry, 2004; Sørensen et al., 2010; Popper et al., 2011; Fangel et al., 2012); the evolution of morpho-anatomical characters in particular rely on cell wall modification. Cell division, cell expansion and cell differentiation, which give rise to the generation of cell shape and plant form, are intrinsically cell wall processes (Szymanski and Cosgrove, 2009; Boudaoud, 2010). For example plant cell division necessitates coordinated synthesis and deposition of a new wall between the two daughter cells and turgor-driven cell expansion depends on wall relaxation mediated for example by enzymes, such as xyloglucan endotransglucosylase (Fry et al., 1992; Nishitani and Tominaga, 1992), or proteins, such as expansins (McQueen-Mason et al., 1992; McQueen-Mason and Cosgrove, 1995), whose presence and action is dependent on wall composition (Franková and Fry, 2011).

Although initially highlighted by biochemical analyses our understanding of the taxonomically-based diversity of plant and algal cell wall components and their biosynthesis has been revolutionized by the availability of sequenced plant genomes (Yin et al., 2009; Popper et al., 2011). There are currently around forty fully sequenced plant and algal genomes (Goodstein et al., 2012). However, in sharp contrast to the late nineteenth century pteridomania which endangered some species (Dyer et al., 2001) ferns (here delimited as the monilophytes which includes ferns, whisk ferns and horsetails) now receive comparatively less attention than many other plant groups and full sequences of fern genomes are, as yet, unavailable (Barker and Wolf, 2010). Cronk (2009) noted that early genome sequencing focussed heavily on angiosperms; perhaps unsurprisingly given their economic prominence. Recently the need for greater phylogenetic coverage has been recognized and, aided by technological advances, has led to the sequencing of representatives of algae and earlier diverged land plants with phylogenetic significance and possessing relatively small genomes including the green alga, *Ostreococcus tauri* (Derelle et al., 2006) and the spike moss, *Selaginella moellendorffii* (Banks et al., 2011). Despite being hampered by its exceptionally large genome size (Burleigh et al., 2012) at ~150 times greater than that of *Arabidopsis*, the first gymnosperm genome, Norway spruce (*Picea abies*), was published earlier this year (Nystedt et al., 2013). Thus, the remaining gaps include sequenced genomes of a fern (Nakazato et al., 2006) and a hornwort (Cronk, 2009). Similarly to gymnosperms, ferns include plants of significant commercial, economic and ecological value such as the aquatic giant salvinia (*Salvinia molesta*) that was recently added to the International Union for Conservation of Nature (IUCN) worst invasive alien species list (Luque et al., 2013). Ferns have a worldwide distribution and are adapted to diverse habitats; often occurring as pioneer species and occasionally becoming ecologically dominant e.g., *Pteridium aquilinum* (commonly known as bracken). Additionally, although ferns consist of ~15,000 species and therefore comprise only around 3% of vascular plant diversity globally (Schuettpelz and Pryer, 2008) they may account for up to 20% of vascular plant diversity in areas such as the West Indies (Groombridge, 1992).

Given the ecological importance and placement of ferns as early diverging euphyllophytes (a sub-division of vascular plants including monilophytes and seed plants) a better understanding of their cell wall complexity, in terms of composition, biosynthesis and tissue- and cell-specific variation, may provide novel insight into key developmental processes, for example vascularisation of leaves (Cronk, 2009), as well as providing unique opportunity to investigate gametophyte-specific processes. In this perspective we review the current state of knowledge regarding fern cell wall composition, the impact of genome sequencing on our understanding of evolutionary pathways of cell wall biosynthetic genes, the requirement for a sequenced fern genome and how this might impact future research focussed on plant cell wall biology, physiology, evolution and development.

## FERN CELL WALLS

**FIGURE 1:**
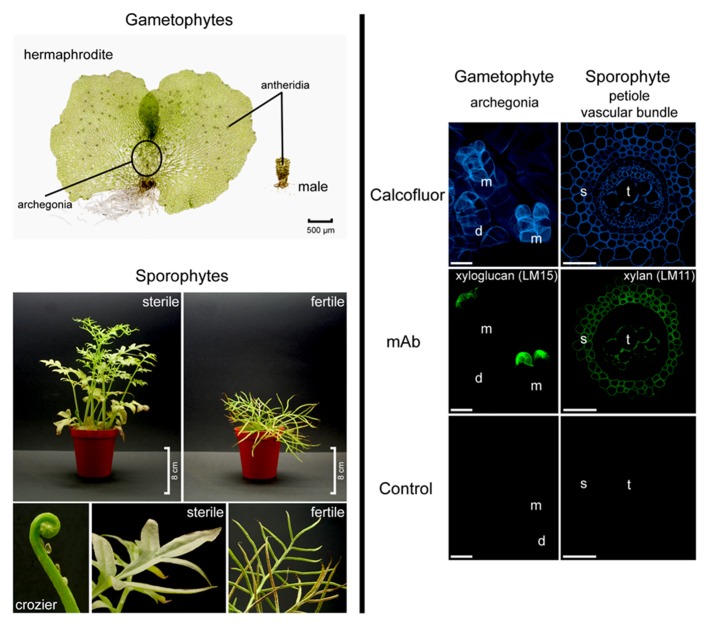
***Ceratopteris richardii* morphology (left hand side).** Gametophytes develop as hermaphrodites or males. Sporophyte fronds are dimorphic. Fronds are initially sterile and oval shaped to three-lobed but new fronds become progressively larger and more pinnately dissected. Fertile fronds are more finely dissected and their enrolled margins are covering the sporangia. **Developmental and tissue-specific variation in *Ceratopteris richardii* cell walls (right hand side).** Localization of cell wall components in hermaphroditic gametophytes and in transverse sections of sporophytic petioles. Calcofluor white stains β-glucans such as cellulose, which occurs in most cell walls. A xyloglucan epitope (mAb LM15) is detected in the apical neck cells of fully mature (and opened) archegonia. LM11, a mAb directed against xylan labeled secondary cell walls of the petiole. d, developing archegonium; m, mature and opened archegonia; mAb, monoclonal antibody; s, sclerenchyma; t, tracheids. Scale bars = 20 μm.

Biochemical analyses have contributed much of what we know about fern cell walls and indicate that they are compositionally similar, though not identical, to those of flowering plants. More specifically, mannose-containing polysaccharides such as mannan and glucomannan appear to be abundant in ferns, whereas pectins appear to be present in lower concentrations than found in other plants (Popper and Fry, 2004; Silva et al., 2011). On the other hand, some wall components have a structure and function which appears to pre-date the divergence of ferns from gymnosperms and flowering plants. α-Expansins, wall-acting proteins which mediate acid-induced wall creep (McQueen-Mason et al., 1992; McQueen-Mason and Cosgrove, 1995), have not only been identified from the ferns *Marsilea quadrifolia* and *Regnellidium diphyllum* (both species of aquatic ferns) by their homology to flowering plant α-expansins but protein extracts from *M. quadrifolia* are capable of inducing wall creep in cucumber cell walls (Kim et al., 2000). The importance of cell wall composition and metabolism to plants environmental responses and survival, as well as our exploitation of them, deem wall composition worthy of extensive exploration. Current approaches include application of specific cell wall-directed tools and methodologies (Fry, 2000; Popper, 2011) including carbohydrate microarrays (Moller et al., 2007), glycome profiling (Pattathil et al., 2012) and microscopy utilizing wall-directed monoclonal antibodies (mAbs), as exemplified in **Figure 1** (right hand side), and carbohydrate-binding modules (CBMs; Sørensen et al., 2009; Pattathil et al., 2010; Hervé et al., 2011) as well as comparative genome analysis.

## THE LYCOPHYTE-EUPHYLLOPHYTE DIVIDE

The genes responsible for the biosynthesis of plant cell wall components are increasingly well identified and characterized. However, the genes responsible for the synthesis and metabolism of some components are not yet fully elucidated (Harholt et al., 2012). This is particularly true for seemingly anomalous occurrences of specific wall components. For instance, although cellulose synthase-like (CSL) supergene family members *CslF*’s and *CslH*’s are responsible for synthesizing β-(1,3)(1,4)-glucan (mixed linkage glucan, MLG) in members of the Poales (grasses; Richmond and Somerville, 2000; Burton et al., 2006, 2008; Doblin et al., 2009) the absence of orthologues of these genes (Harholt et al., 2012) confounds detection of MLG in *Selaginella* *moellendorffii* and synthesis of MLG in this plant remains enigmatic but is corroborated by the discovery of MLG in *Equisetum* (horsetails; Fry et al., 2008; Sørensen et al., 2008)).

Sequencing and genome analysis of the whisk fern *Selaginella moellendorffii*, chosen for its small genome size (Banks et al., 2011; Harholt et al., 2012), has already proven invaluable to elucidating diversification of cell wall components and their biosynthetic machinery (Popper et al., 2011). Lycophytes are the earliest diverging extant plants to have a vascular system and a dominant sporophyte generation. However, disparities in genome sequence and cell wall biochemistry between *Selaginella* and other sequenced vascular plants including *Arabidopsis* (Arabidopsis Genome Initiative, 2000), *Populus* (Tuskan et al., 2006), and the grasses, rice (International Rice Sequencing Project, 2005), and *Brachypodium* (International Brachypodium Initiative, 2010), detailed below, highlight the need for fern sequences and detailed cell wall studies, not only to help better understand ferns, but also euphyllophyte evolution and development.

Although the majority of cell wall components found in flowering plants also occur in *Selaginella*, Harholt et al. (2012) observed differences in the abundance, localization and extractability between wall polymers in flowering plants compared with those in *Selaginella*. This is potentially indicative of differences in interactions between specific cell wall components. Pectins in particular appeared to not only be more abundant in lycophytes than in angiosperms but also required harsher extraction procedures (Harholt et al., 2012). The pectin, rhamnogalacturonan II, was found to occur in all vascular plant groups in similar concentration but, despite appearing to be highly conserved, exhibited a minor compositional variance; in lycophytes, ferns and whisk ferns; a rhamnose residue was replaced by a 3-*O*-methyl rhamnose residue in one of the side chains (Matsunaga et al., 2004). Furthermore, some cell wall features appear to have arisen through convergent evolution. For example the regulation and biosynthesis of syringyl (S) lignin which reinforces the secondary cell walls in the vascular tissue of flowering plants and lycophytes, but is absent from the majority of ferns and gymnosperms, occur via independent pathways (Weng et al., 2008, 2011; Zhao et al., 2010; Novo-Uzal et al., 2012). In angiosperms S lignin is synthesized from guaiacyl lignin intermediates by ferulic acid/coniferaldehyde/coniferyl alcohol 5-hydroxylase (F5H) and although *Selaginella moellendorffii* contains a functional F5H it is not orthologous to angiosperm F5Hs instead belonging to a clade of genes unique to *Selaginella* (Weng et al., 2008). As Harholt et al. (2012) point out this is in direct contrast with an apparent lack of diversification and specialization within the cellulase synthase (CESA) superfamily. Homologs of IRX10, also involved in vascular formation in land plants, were found in the moss *Physcomitrella patens* and were recently reported to exhibit functional conservation with those from *Arabidopsis* (Hörnblad et al., 2013). Taken together these data suggest that at least some components of vascular tissues considered to be a “hallmark” of vascular plants (Weng et al., 2008), are not homologous between the lycophyte and euphyllophyte vascular plant lineages. Lycophytes also have unique primary cell wall characters. The isolation of uniquely high concentrations of the unusual sugar residue 3-*O*-methyl-D-galactose had previously been considered an autapomorphy of the lycophytes as its occurrence was restricted to homosporous (including *Lycopodium pinifolium*, *Huperzia selago,* and *Diphasiatrum alpinum*) and heterosporous lycophyte (including three species of *Selaginella* although not *S. moellendorffii*) primary cell walls (Popper et al., 2001).

Despite accounting for only 5–10% of the dry mass of cell walls (Jamet et al., 2008) proteins are intrinsically responsible for wall synthesis, structure and function, primarily through their modification of other cell wall components, such as polysaccharides, in response to developmental and environmental cues. There appears to be a phylogenetic basis to the profile of cell wall-acting enzymes possessed by a specific plant. While some enzyme activities, such as xyloglucan endotransglucosylase, which coordinates expansive plant cell growth by cutting and rejoining of intermicrofibrillar xyloglucan chains (Fry et al., 1992; Nishitani and Tominaga, 1992), appear to be present in all vascular plants (Vissenberg et al., 2003) others show a disjuncture between lycophytes and euphyllophytes. Franková and Fry (2011) extracted and assayed proteins from 57 rapidly growing plant organs from a range of flowering plants, *Selaginella* (*apoda*), a horsetail and a liverwort and found remarkable differences in glycanase (endo-hydrolase) and glycosidase (exo-hydrolase) activities which correlated with differences in wall composition. For instance, β-mannosidase activities were highest in plants with mannan-rich endosperms requiring rapid metabolism during germination rather than in plants, including *Selaginella*, whose vegetative tissues have mannan-rich cell walls (Franková and Fry, 2011). Polygalacturonases (PGs) are a large family of hydrolytic enzymes (Kim et al., 2006) which modify pectins developmentally. Analysis of *Arabidopsis*, *Populus*, rice, *Selaginella,* and *Physcomitrella* genomes indicate an expansion of the PG gene family occurred after the divergence of the lycophytes and euphyllophytes; 16 PG genes were identified in the lycophyte *Selaginella* in comparison with 44 in rice and 75 in *Populus* (Yang et al., 2013). Although lycophytes and euphyllophytes have shared characteristics including vascular tissue and a dominant sporophyte generation they last shared a common ancestor 400 million years ago (Pryer et al., 2004) and there are many differences as summarized in **Table 1**. A fundamental difference between the groups is that lycophytes possess microphylls whereas euphyllophytes possess structurally more complex, particularly with respect to vascularisation, megaphyll leaves (Cronk, 2009). The two organs appear to be developmentally and morphologically distinct which, in combination with the existence of many leafless but otherwise highly complex fossils, has led to relative consensus that despite having similar functional roles microphylls and megaphylls are not homologous (Cronk, 2009). Yang et al. (2013) hypothesized that expansion of the PG gene family may be correlated with the evolution of leaves and increased organ complexity but emphasized that the current sample of sequenced vascular plant genomes, which does not yet include ferns, does not enable dating of the PG gene family expansion. However, spatial-temporal changes in remodeling of cell wall components, such as pectins by PGs, lead to changes in wall biomechanical properties, resulting in altered development and morphology (Boudaoud, 2010).

**Table 1:** Summary of differences between the lycophyte *Selaginella moellendorffii*, fern *Ceratopteris richardii*, and angiosperms.

Character	*Selaginella moellendorffii*	*Ceratopteris richardii*	Flowering plants
Taxonomic grouping	Lycophyte	Fern	Angiosperms
Ploidy of sporophytes	Diploid	Diploid	Various
Dominant generation	Sporophyte	Sporophyte	Sporophyte
Gametophytes	Endosporic (remain largely enclosed in spore tissue), subterranean	Exosporic and photosynthetic	Endosporic (remain enclosed in sporophyte tissues)
Primary photosynthetic organ	Microphylls, typically with only a single unbranched vascular strand	Megaphylls (euphylls), lateral organs of the shoot, derived from stems and possessing branched vasculature	Megaphylls (euphylls)
Plant axis	Rhizophore, homorhizic roots (which develop laterally relative to the embryonic axis of the embryo), and stem	Homorhizic roots, and stem	Allorhizic roots (which develop at the opposite end of the embryonic axis to the shoots (eudicots), or a secondarily homorhizic root system (most monocotyledonous plants), and stem
Mega- and micro-sporangia	Heterosporous, typically producing four megaspores in the megasporangium and hundreds of micro-spores in the micro-sporangium	Homosporous, producing hermaphrodite and male gametophytes	Heterosporous, producing a dispersed ovule (mega-sporangium protected by an integument)
Branching pattern	Dichotomous (derived from dichotomous branching of the shoot apical meristems)	Lateral	Lateral

As outlined above the distinct differences in cell wall biochemistry between lycophytes and euphyllophytes is perhaps not unexpected because lycophytes are distinguished as a sister group to all other vascular plants with associated key differences in anatomy and development (Judd et al., 1999; Pryer et al., 2001; Banks, 2009). Therefore, a model fern may provide key insight into whole plant development (Tilney et al., 1990; Racusen, 2002) and the impact of cell wall metabolism.

## C-FERN CELL WALLS

A strong foundation for using *Ceratopteris richardii*, often referred to as C-Fern, as a model to investigate the influence of cell walls on development has been laid by anatomical and cytochemical investigations. Such studies include scanning electron microscopy of xylem (Carlquist and Schneider, 2000), gametophyte development (Banks, 1999), embryogenesis (Johnson and Renzaglia, 2008), the histology of spermatocyte cell wall composition (Cave and Bell, 1973) and drug-induced perturbation of cellulose synthesis in root hairs (Meekes, 1986). The latter study indicated that C-Fern responds to cell wall-acting drugs in a similar way to flowering plants. Additionally C-Fern is highly sensitive and provides opportunity to investigate drug action; in a single cell layer, in free-living haploid tissues (gametophytes), and in combination with microtubule organizing centers which might be important in order to investigate the effects of microtubule disruption on cell wall component secretion (Meekes, 1986). Furthermore, an array of C-Fern mutants exists including some that may have altered cell walls. One of the most striking is polka dot, which has clumped chloroplasts, putatively resulting from disruption to the cytoskeleton (Vaughn et al., 1990), which may have led to the observed associated weaknesses in spore walls.

## C-FERN AS A MODEL PLANT

Clearly, as previously voiced by others (Weng et al., 2008; Cronk, 2009), there is a requirement for sequenced fern genomes. Although there are currently no fully sequenced fern genomes the National Center for Biotechnology Information’s (NCBI) short read archive (SRA) database has incomplete genome data for two ferns, *Ceratopteris richardii* and, the perhaps more universally familiar invasive, *Pteridium aquilinum* (). The *Pteridium* sequence is derived from a gametophyte transcriptome (Der et al., 2011) similarly the C-Fern expressed sequenced tags (ESTs) are from the early stages of development in germinating spores (Salmi et al., 2005); both sequences are therefore equivalent to the tissues which give rise to pollen grains and embryo sacs in flowering plants. Curiously although wall synthesis and restructuring are required for gametophyte development, particularly cell division and expansive cell growth, less than 1% of the gene products expressed in *Ceratopteris* spores are cell wall-localized (Salmi et al., 2005). Since annotation was carried out by BLAST comparison with the *Arabidopsis* genome one possibility is that fern and flowering plant cell wall-localized genes are significantly divergent.

Leptosporangiate ferns, of which *Ceratopteris richardii* and *Pteridium aquilinum* are members, comprise over 95% of extant fern diversity (Schuettpelz and Pryer, 2008). In fact both of the aforementioned species belong to the polypods, a clade strongly supported by molecular and morphological characters including sporangia which possess a vertical annulus interrupted by the stalk (Pryer et al., 2001; Schuettpelz and Pryer, 2008). However, whereas *Pteridium* is placed in the small dennstaedtioid clade, *Ceratopteris* belongs to the large, diverse pteridoid clade which accounts for about 10% of extant fern diversity (Schneider et al., 2004; Schuettpelz and Pryer, 2008); this suggests that *Ceratopteris* is likely to be highly representative of other ferns. *Ceratopteris* is homosporous and produces hermaphrodite and male gametophytes (see **Figure 1**). The male gametophytes are produced in response to antheridiogen (Schedlbauer and Klekowski, 1972). The diploid sporophytes are extremely heteroblastic, initially producing entire sterile leaves and progressing to highly dissected fertile leaves which, under culture conditions, produce many spores continuously throughout the year within sporangia on their enrolled leaf margins (Hickok et al., 1987; **Figure 1**, left hand side). In comparison to many other ferns including *Pteridium*, * Ceratopteris* sporophytes are relatively small, reaching 30–40 cm in height. This feature particularly coupled with its ease of growth in culture has been responsible for the widespread application of *Ceratopteris* as model in undergraduate plant biology teaching, for example to demonstrate plant lifecycles, genetics and development, and in research laboratories (Hickok et al., 1987, 1998; Calie, 2005; Spiro and Knisely, 2008). This has lead to the development of specific tools and techniques including mutant generation, selection and characterization; mutants include abscisic acid (Hickok, 1985), herbicide-tolerant (Hickok and Schwarz, 1986) and salt-tolerant (Warne et al., 1995). Other features which make *Ceratopteris *a suitable model include: (1) a short sexual life cycle which can be completed in under 120 days, (2) continuous and abundant spore production, (3) spores that can be stored and remain viable for many years, (4) gametophytes which can be self-fertilized to generate completely homozygous sporophytes, (5) visible microtubule organizing centers and developmental synchrony of cells within a single gametophyte (Hoffman and Vaughn, 1995), (6) sporophytes that can be vegetatively propagated from marginal leaf buds or gemmae allowing maintenance of even sterile mutants (Hickok et al., 1987) and (7) amenability to mutagenesis. Furthermore, although experiments initially suggested that *Ceratopteris* is resistant to *Agrobacterium*-mediated transformation (Hickok et al., 1987) *Agrobacterium* has now been shown capable of transforming *Ceratopteris thalictroides* (and Chinese brake fern, *Pteris vittata*) spores leading to stably transformed plants; inheritance analyses revealed stable expression of the transgene in second generation sporophytes (Muthukumar et al., 2013). Additionally, *Ceratopteris* gametophytes have been shown to take up DNA and RNA directly enabling elucidation of gene function through observation of phenotype following targeted silencing (Stout et al., 2003; Kawai-Toyooka et al., 2004; Rutherford et al., 2004).

## LOCATION, LOCATION, LOCATION AND FUTURE PERSPECTIVE

Although a fully sequenced fern genome will be available in the near future, likely contributing much to our understanding of the evolution of euphyllophytes, plant cell wall components and their biosynthesis, it is unlikely to reveal the full story. The reason for this is that many wall components are deposited in a tissue, cellular or even sub-cellular fashion, often in response to development (Leroux et al., 2007, 2011). Therefore, genomic studies will yield most information when carried out in combination with localization of wall components using (immuno)cytochemical methods (Cave and Bell, 1973; Hervé et al., 2011). Many of the mAbs and CBMs developed to flowering plant cell walls have the ability to recognize and bind to epitopes present in bryophyte (Carafa et al., 2005) and fern (Leroux et al., 2007, 2011) cell walls including those of C-Fern (as exemplified by **Figure 1**). The ability to apply these techniques to *Ceratopteris* (and other ferns) provides advantages for investigating plant development involving the cell wall, not afforded by earlier diverging vascular plants. For example *Selaginella* gametophytes are endosporic, meaning that the female gametophyte remains enshrouded in spore tissue, and subterranean. Flowering plant gametophytes are similarly embedded in sporophyte tissues. In contrast fern gametophytes which are photosynthetic and free-living can be prepared (relatively) easily for biochemical analysis. Furthermore, it is possible to follow every cell throughout development. A fern model, such as *Ceratopteris*, once sequenced will build on what has already been uncovered by investigation of other sequenced plants, particularly other vascular plants such as *Selaginella*, and likely divulge many secrets relating to euphyllophyte cell wall biochemistry, evolution and function.

## Conflict of Interest Statement

The authors declare that the research was conducted in the absence of any commercial or financial relationships that could be construed as a potential conflict of interest.
